# Medical and Physical Intelligence

**Published:** 1802-07

**Authors:** 


### MEDICAL AND PHYSICAL INTELLIGENCE.

### A SHORT AND CONVENIENT METHOD OF PREPARING EMETIC TARTAR.

Mr. BUCHHOLZ, an able apothecary, at Erfurt, has made ſeveral experiments concerning the moſt eaſy mode of obtaining Emetic Tartar. It is well known how tedious the common method of boiling the glaſs of antimony and the cryſtals of tartar is, and that an eaſier and more expeditious method was much wanted. When he occaſionally mentioned this circumſtance to one of his colleagues, he was informed that there was little difficulty in diſſolving the glaſs of antimony in purified cryſtals of tartar, by finely pulveriſing both ſubſtances and mixing them with diſtilled water to the conſiſtency of a pulp like honey; which being ſuffered to ſtand during two or three weeks in a warm place, and ſtirred ſeveral times a day, will be eaſily diſſolved by boiling water. In conſequence of this remark, Mr. B. undertook the following experiment. One ounce of vitrum antimonii being mixed with two of purified cryſtals of tartar, and a ſufficient quantity of diſtilled water to the conſiſtency of a pulp, was expoſed to the heat of the ſun under a glaſs bell, and ſtirred twice or three times a day: freſh water was occaſionally added, in order to keep the mixture of a proper conſiſtence. After being digeſted for twenty-four hours, the whole maſs began to puff up, and to emit the ſmell of ſulphurated hydrogen gas, and ſmall particles particles and flakes, fimilar to Kermes' mineral, appeared, which increafing by degrees, imparted a brown colour to the mixture, and a little portion of it being diffolved in diftilled water and filtrated, difcovered by adding to it a folution of fulphurated kali a confiderable quantity of antimonial particles. After having in this manner treated the above mixture for about a fortnight, during which time the fmell of fulphurated hydrogen gas continued, Mr. B. proceeded to the folution in boiling water, in which the whole mafs nearly diffolved, except a few grains of gravelly particles, and a confiderable quantity of a fubftance fimilar to Kermes' mineral. Befides the common cryftals of emetic tartar, Mr. Buchholz obtained at the firft fhooting of cryftals, a quantity of fmall, brittle, yellowifh cryftals, which feemed to be impregnated with iron. As there was part of the mixture loft, the proportion of the products which were obtained could not be accurately determined. In a fecond experiment, made with the fame quantity of glafs of antimony and cryftals of tartar, the whole mixture, after being digefted for above twelve days, diffolved, except 56 grains of gravelly particles, and others fimilar to Kermes' mineral, which laft fubftance being previoufly burnt, left behind 20 gr. of oxyd of antimony. The whole quantity of falt which was obtained in all the cryftallizations, amounted to about *two* ounces *three* drachms, together with a confiderable quantity of pure tartar in fmall yellowifh cryftals, and fome yellowifh falt crufts: the incryftallizible refiduum was of a greafy confiftence, and of a brown colour.

In order to determine the proportion of glafs of antimony, which may be required for faturating the remaining tartar, and at the fame time to afcertain the quantity of the products, Mr. B. undertook feveral experiments, with the fame and greater portions, the refults of which were the following. It was found, that for thoroughly faturating the whole quantity of purified tartar employed for the operation, the proportion of *one* part and *a half* of vitrum antimonii is required to *two* parts of cryftals of tartar; and from 3 $\frac{1}{4}$ ounces of this mixture, he obtained, according to his experiments, *two* ounces and *three* drachms of cryftallized emetic tartar: the Kermes' mineral, remaining from 12 dr. of vitrum antimonii, amounted to about 1 $\frac{1}{2}$ dr. Befides the cryftals of emetic tartar, he obtained the above mentioned fmall, brittle, yellowifh cryftals, and half a drachm of yellowifh powdery fubftance: the incryftallizible, greafy, brownifh refiduum weighed, when dry, about 70 grains, and received then a greyifh colour. Mr. B. upon examining this refiduum, and the two heterogeneous falts, obtained at the fame time with the cryftals of emetic tartar, found the refiduum to contain tartrite of kali as well perfectly as imperfectly faturated with iron, and a fmall quantity of oxyd of antimony; the whitifh fmall cryftals to be tartrite of lime, &c. With refpect to the folubility of the cryftallized emetic tartar in diftilled water, he found that, at a temperature of 10 to 12° Reaum. 14 $\frac{2}{7}$ parts of diftilled water were requifite for diffolving *one* part of emetic tartar, which proportion greatly differs from what is commonly believed, viz.

viz. that 80 parts of water are requiſite for one part of emetic tartar.

At 80° Reaum. 100 parts of diſtilled water diſſolved 53 parts of cryſtallized emetic tartar.

Pure emetic tartar ought to be quite white, as any admixture of yellowiſh colour proves its being impregnated with iron. The principal reſult of all theſe experiments is, that the ſolution of the vitrum antimonii by pure tartar may be moſt eaſily performed, by mixing them together to the conſiſtence of pulp, which is ſuffered to ſtand in a warm place, and the thus generated emetic tartar may be ſeparated by the mere addition of boiling water, in which manner much time and expence will be ſaved, and a large apparatus rendered unneceſſary, which is generally uſed.

---

Mr. Sebald, veterinarian of Ulm, has had an opportunity of diſcovering a great quantity of ſtones in the inteſtinal canal of a miller's horſe. This horſe had ſeveral days laboured under a cholic, with an obſtinate obſtruction of the belly, which was tumid and tenſe; neck and ſides were covered with a cold ſweat, the pulſe very frequent, reſpiration quick and ſhort, and the ſlighteſt motion threatened to ſuffocate it. Notwithſtanding all poſſible remedies, the horſe died in three days; and on opening it, one hundred and thirty-four ſtones, of different ſize and figure, were found diſperſed in the inteſtinum coecum and colon. The largeſt of them, lying in the inteſtinum coecum, was the ſize of a middling bowl, poliſhed, and of a dark colour; its weight, however, amounted only to about 4lb. and a common calculus of the ſame ſize would have weighed three times as much. The ſecond ſtone was as large as a man's fiſt, of a rough and uneven ſurface, and weighed about 1lb. 8 ounces and a half. It ſtuck in the colon, where it had cauſed a topical inflammation, and perforated the inteſtine, but without falling into the cavum abdominis; and it is probable, that it was the principal cauſe of the animal's death. The different ſtrata of which it was compoſed, ſhowed its progreſſive formation. The third ſtone was ſituated between the two former, of the ſize of a middling apple, and weighed about 11 ounces 3 drachms; the remaining 131 ſtones, of different ſize and figure, were mixed with the excrements in the inteſtinum coecum, and had a kernel in their middle: they weighed together about five ounces and a half. There is no doubt but that the formation of theſe ſtones may be aſcribed to the gravelly particles rubbed from the grindſtone and mixed with the bran with which the horſes of millers are generally fed. Similar inſtances of ſtones being found in ſuch horſes are not uncommon; and Mr. Sebald remembers to have ſeen a ſtone of this kind, weighing 7lb. and another of 13lb. but he does not recollect any caſe where ſtones were found in ſuch great quantity, and of ſo different ſize and figure, as in this caſe. It were to be wiſhed, that a chemical analyſis might be made of theſe calculous concretions of the inteſtinal canal.

---

Profeſſor

Profeffor Osiander of Gottingen, in his Annals of the Gottingen Lying-in Hofpital, (Vol. i. No. 1, in German), mentions a cafe of a delivery, whereby he obferved *a pulsation in the vessels of the umbilical cord and placenta,* which continued for a confiderable time after the delivery. The fecundinæ followed a few moments after the child came forth, a circumftance, which allowed him to make the following obfervation. The child being placed in a fmall trough, with warm water, and likewife the placenta, which was not yet feparated, into another veffel ftanding next to it, the arteria umbilicalis began vifibly to beat at the exterior and interior fide of the placenta, which continued for nearly 25 minutes fo ftrongly, as to put the placenta into confiderable motion: but the moft remarkable circumftance is, that not the leaft drop of blood iffued from the placenta, which evidently proves, that no blood paffes over from this organ into the maternal veffels, commonly thought to be inofculated in the placenta, but that the circulation of blood in the fetus takes merely place between the placenta and the fetus. Nor does there feem any blood driven from the uterus to the placenta, as well as vice verfa; and it is more probable, that the fuperfluous parts of the infantile blood are equally abforbed by the veins and lymphatics of the uterus, as the parts from which the darkened blood in the fetus is prepared, are reforbed by the lymphatic veffels of the placenta from the uterus. In another cafe it was obferved, that the pulfe in the umbilical cord beat 126 times in a minute.

---

We lament to have occafion to announce the premature death, on Monday, June 28, of Dr. Thomas Garnett, late Profeffor of Natural Philofophy in the Royal Inftitution, and celebrated as a Public Lecturer in this metropolis He was a man the qualities of whofe mind fecured him the love of all who knew him, and who was poffeffed of extenfive learning, of accurate obfervation, and of eminent qualifications as a Phyfician and Lecturer in Philofophy. He was fuddenly cut off in his 38th year, by a typhus fever, which he had caught in his practice as Phyfician to a Difpenfary, to which he had been but a few weeks appointed.

---

*Extract of a Letter from Constantinople, dated April* 26, 1802.

"Dr. Heffe, whofe indefatigable zeal diffufes and keeps up, in this capital, the infertion of the Vaccine matter, is remunerated for his exertions by the ample fuccefs he experiences.

"Several children who were inoculated laft fummer, have been fent among thofe who were labouring under the Small-pox, for the purpofe of inhaling their breath; others have been regularly inoculated without experiencing the moft remote influence of the contagion. In fhort, all the children whom Doctors Heffe, Scott, and Pezzoni inoculated for the Cow-pox in different villages of the vicinity, particularly thofe of Belgrade and Bonyonkdere, have been exempt from the fury of the Small-pox, which have raged with fuch uncommon violence during the whole courfe of the laft winter.

"It

"It is a pleaſing conſideration, that the Kiſmet, or the ſyſtem of predeſtination, which has always acted ſo powerfully on the minds of the Turks, as to make them reject every idea of inoculation, which the Greeks adopted with enthuſiaſm, has been overturned in favour of the Vaccine Inoculation. Some of the moſt diſtinguiſhed families of the Ottoman Empire have had their children inoculated!"

---

